# Privacy versus Public Health? A Reassessment of Centralised and Decentralised Digital Contact Tracing

**DOI:** 10.1007/s11948-021-00301-0

**Published:** 2021-03-29

**Authors:** Lucie White, Philippe van Basshuysen

**Affiliations:** Leibniz Universität Hannover (Institut für Philosophie), Hannover, Germany

**Keywords:** COVID-19, Digital contact tracing, Privacy, Efficacy, Risk, Data ethics

## Abstract

At the beginning of the COVID-19 pandemic, high hopes were placed on digital contact tracing. Digital contact tracing apps can now be downloaded in many countries, but as further waves of COVID-19 tear through much of the northern hemisphere, these apps are playing a less important role in interrupting chains of infection than anticipated. We argue that one of the reasons for this is that most countries have opted for decentralised apps, which cannot provide a means of rapidly informing users of likely infections while avoiding too many false positive reports. Centralised apps, in contrast, have the potential to do this. But policy making was influenced by public debates about the right app configuration, which have tended to focus heavily on privacy, and are driven by the assumption that decentralised apps are “privacy preserving by design”. We show that both types of apps are in fact vulnerable to privacy breaches, and, drawing on principles from safety engineering and risk analysis, compare the risks of centralised and decentralised systems along two dimensions, namely the probability of possible breaches and their severity. We conclude that a centralised app may in fact minimise overall ethical risk, and contend that we must reassess our approach to digital contact tracing, and should, more generally, be cautious about a myopic focus on privacy when conducting ethical assessments of data technologies.

## Introduction

Contact-tracing apps were initially heralded as the key to keeping the spread of COVID-19 under control, but this promise has been all but abandoned, with governments now downplaying the potential efficacy of the measure, and suggesting that it will have, at best, a limited role among a host of other mitigation measures (Lomas, 2020b; Taylor, 2020). We argue that this is partially due to the specific “decentralised” configuration of the app (that is, a configuration in which most information is stored on the user’s own phone rather than on a central server) that has been adopted in many countries, which, after a debate in which the voices of privacy advocates featured strongly, has come to be largely regarded as the ethically superior alternative, because it is “privacy preserving by design”. We contend, on the contrary, that an app with a different configuration, namely, an app that stores some pseudonymised information on a central server (that is, a “centralised” app), and that allows for reporting before a confirmed test, shows promise in increasing the efficiency of the measure.

Once this is clear, it also becomes apparent that we must widen our focus beyond privacy concerns. Rather, we might be thought to be faced with a sort of trade-off of ethical risks: with risks of privacy infringements on the one hand, and risks of impairing efficacy, and thus of forgoing public health benefits, on the other. We argue that, rather than being privacy preserving by design, decentralised systems entail risks of breaches too. Then, drawing on principles from safety engineering and risk analysis, we compare the risks of centralised and decentralised systems along two dimensions, namely the probability of possible breaches and their severity. In order to make up for the higher probability of achieving public health benefits that centralised systems can provide, decentralised systems would need to exhibit considerable advantages on at least one of these dimensions, which we argue is not the case. Thus, once we understand the type of ethical trade-off that must be conducted here, we can see that the centralised app may indeed be ethically preferable. Our proposed approach for assessment provides a way of incorporating, but also looking beyond, privacy concerns in the evaluation and assessment of data technologies. The case of digital contact tracing in the COVID-19 pandemic presents us with a cautionary tale against letting privacy concerns dominate debates on data technologies, and overall risk analysis provides a potential means of widening our scope to encompass and evaluate other ethical concerns.

## To Increase Its Chances of Being Effective, Digital Contact Tracing Requires Centralised Data Storage

There is, by now, consensus that two factors are key if a contact-tracing app is to make a significant impact on viral spread: it will need sufficiently high uptake, and it will need to allow for very fast intervention (i.e. persons that are likely to be infected must be identified and quarantined very quickly) (Braithwaite et al., 2020; Kretzchmar et al., 2020). There has been some discussion of various ways in which *uptake* might be increased (see e.g. Hernández-Orallo et al., 2020; Loi, 2020). We will focus here on increasing the efficiency of the app through increasing the *speed* at which contacts can be identified and quarantined.^1^ There is an obvious way to do this. At present, digital contact-tracing systems require that persons receive a positive PCR test before reporting on the app that they are positive for COVID-19 (which then results in an alert to those who they have been in high-risk contact with, advising them to self-quarantine and/or get tested) (Ahmed et al., 2020).^2^ The process could be sped up significantly if people could report that they might be infected immediately upon experiencing potential symptoms. This is particularly essential for COVID-19, because it appears that individuals become infectious shortly after they themselves are infected, and that a substantial degree of virus transmission occurs before the onset of symptoms (Ganyani et al., 2020; He et al., 2020). Allowing for reporting directly at symptom onset would allow contacts to be alerted to quarantine before they have begun to experience symptoms, thus isolating them before they are well into their window of infectiousness. Indeed, some mathematical modelling suggests that “delaying contact tracing by even half a day from the onset of symptoms can make the difference between epidemic control and resurgence” (Hinch et al., 2020). No matter how quickly PCR testing can be conducted, it seems very difficult to imagine that tests can be routinely sought, administered, the results received, and reported in the app within this small window.

Given the agreement that speed is of the essence here, how did it come about that such a configuration has not been implemented? We suggest that this is largely due to the development of the debate on contact tracing apps. Such a system, we will show, requires that some information is stored on a centralised server. But the early debate on contact tracing apps quickly became dominated by privacy concerns. Privacy advocates argued that the centralised storage of information entailed the unacceptable risk of privacy breaches, and that an app configuration in which most information was stored in a decentralised manner (i.e. on the user’s own smartphone) is “privacy preserving by design” and thus ethically superior (see e.g. Joint Statement, 2020; Lomas, 2020a; Troncoso et al. 2020). The original proposal to store information on a centralised server, and thus allow for reporting before a test, was made by Ferretti et al. (2020) and Hinch et al. (2020), and was originally used as the basis for the UK’s centralised contact tracing app. However, as privacy advocates continued to make a stand against the centralised storage of information, Apple and Google announced that they would only support governments developing decentralised apps, providing them with the toolkit to accurately detect contact events, and to allow the app to run in the background while users go about their daily business (Scott et al. 2020). After persevering for a while with their centralised app, the UK was ultimately unable to independently solve these technical problems, and abandoned their centralised approach in favour of the decentralised option that could meet Apple and Google’s requirements for cooperation, while at the same time beginning to downplay the importance of digital contact tracing altogether (Lomas, 2020b). Other countries, such as Australia, Singapore (Criddle & Kelion, 2020) and Germany (Scott et al., 2020) also considered or pursued a centralised app before switching to a decentralised configuration to work with Apple and Google.

Before we turn to the concerns of privacy advocates that so shaped the trajectory of contact-tracing apps during this pandemic, we will briefly outline why the storage of some information in a centralised manner is necessary to allow for rapid reporting. First, we will need to get into the fundamentals of how contact-tracing apps work. Most contact tracing apps work on the basis of Bluetooth signals, which are used to gauge when two people (or, at least, their phones) come into close contact, and for how long. Each person is assigned a frequently-changing series of ID-numbers (“ephemeral identifiers”). When two people come into proximity, their phones exchange ephemeral identifiers via Bluetooth. When someone reports that he is positive for COVID-19 on the app, anyone who has this person’s ephemeral identifiers on her phone during the estimated window of infection can be immediately alerted and sent into quarantine.

The difference between a decentralised and centralised app configuration, as already mentioned, inheres in where information is stored. In a decentralised app, the ephemeral identifiers are created and stored on each individual user’s smartphone. The central server only comes into play when a user reports as positive—in this case, his own ephemeral identifiers for the period of infection are uploaded to a central server and then broadcast to all app users, who are then alerted when one of these identifiers is stored on their phone. In a centralised app configuration, the central server plays a larger role: each user is assigned a *permanent pseudonymous identifier*, which is stored on the central server. Ephemeral identifiers are created on the server, and sent to each user’s phone. Phones exchange ephemeral identifiers, and when a user reports as positive, the ephemeral identifiers of his contacts are sent to the server, which matches these to their permanent identifiers and alerts the corresponding contact (Vaudenay, 2020).

It is this storage of a permanent identifier that allows centralised contact tracing apps to accommodate reporting before a test—because this provides a way of dealing with false positive reports. As many may have anticipated, this will clearly be a problem when users can submit reports before a confirmed infection. The symptoms for COVID-19 can be difficult to identify, leading to the possibility that many positive reports might arise from genuine mistakes. There is also the possibility that some users might submit malicious false reports in order to disrupt the system. One possibility for mitigating the impact of false reports could be to require that reports are followed up with a positive test within a certain period of time—contacts could be temporarily quarantined, and then released, say, three days later if no follow-up test is forthcoming. The problem with this strategy is it is likely to break down completely when there is any delay accessing a test, or any shortage of tests. It also requires that a sufficient amount of users are diligent enough to follow up their report by immediately seeking a test and reporting their results. If any of these conditions are not met, either the quarantine period must be extended to allow time for a test to be sought, conducted and reported (leading to longer periods of erroneous quarantine), or contacts must simply be released where no follow-up is forthcoming (which could lead to the release of too many true positive cases, hampering the effectiveness of the measure).

However, there is an alternative way to identify false positive reports, contingent on the ability to identify clusters of cases. When a certain proportion of an index case’s contacts subsequently contract the virus, we can identify a cluster. When none (or few, depending on the background rate of infection) of an index case’s contacts subsequently become infected, this might indicate that the initial report was a false positive (Hinch et al., 2020). This can proceed on a centralised app by following contacts over time. The server in a centralised app has enough information to determine, on the basis of the permanent identifiers of users, whether an initial report is followed by subsequent reports from contacts (and how many, and the duration and proximity of the contact). This provides a means of identifying likely false positive reports in the absence of a follow-up test, and a means of releasing users from quarantine. This can all proceed without directly identifying any of the app’s users—permanent pseudonymous identifiers will suffice for this purpose. In a decentralised app, there is no way to identify clusters—each smartphone only holds the ephemeral identifiers of direct contacts, and the server only holds the ephemeral identifiers of infected users for the period of infection. There is no way to track contacts through time and thus identify clusters of infection, or, more to the point, a lack thereof, indicating a likely false positive report.

To summarize, it is clear that speed of contact tracing will be absolutely crucial as a means to stop the viral spread of COVID-19 (or other viruses like it). Even short delays can significantly diminish the effectiveness of this measure. We can speed up the process (from identification of a likely case to quarantine) by allowing people to report as positive on the app directly upon experiencing symptoms of COVID-19. This, however, leads to the problem of false positive reports. We can mitigate this problem, minimising the duration of erroneous quarantines, by identifying where a report of infection is not followed up by (a sufficient number) of subsequent reports from contacts, indicating a likely false positive report, and allowing for the early release of contacts. But this can only be done when the permanent identifiers of app users are stored on a central server, allowing us to track contacts through time.^3^

## Inherent Safety versus Secondary Prevention Measures

Having established that, in order to increase the chances of being effective, digital contact tracing requires the centralised storage of some pseudonymised information concerning each user, we will now turn to a general ethical evaluation of the different contact tracing options. There are various values that should guide the design and implementation of contact tracing apps and ethical challenges that should be met (Lucivero et al., 2020; Ranisch et al., 2020). While many substantive and procedural values, such as justice and transparency, can be expected to be had “for free”, that is, without violating other important values, we will focus here on those that might generate trade-offs that will be crucial for an ethical evaluation: namely, is there a configuration of the app with the ability to achieve the *public health benefits* it is supposed to achieve, while at the same time respecting its users’ *privacy*? Or does the fulfilment of one of these values require a configuration that risks impairing the other?

As we have shown, the centralised storage of data allows us to configure the app in a way that can make it faster and thus more effective, allowing for increased public health benefit (through better interrupting chains of infection). However, advocates of decentralised systems contend that apps for digital contact tracing should be “privacy preserving by design” (Joint Statement, 2020). They argue that this condition can be satisfied by decentralised systems, but not by centralised ones, because the centralised storage of information entails the risk of breaches that, if realised, would infringe on users’ privacy (Joint Statement, 2020; Troncoso et al., 2020). If this argument holds water, we are faced with a stark trade-off between respecting users’ privacy on the one hand, and reaping the public health benefits from an app on the other.

To evaluate the argument, it is instructive to compare this notion of “privacy preserving by design” to a principle from safety engineering, namely *inherent safety*: a design is inherently safe if it eliminates a potential hazard altogether, rather than applying additional safety measures to decrease its risk of being realised (Möller & Hansson, 2008). For instance, making use of fireproof materials is inherently safe while using inflammable materials is not. While the risk of a major fire occurring in the latter case could be reduced by means of a “secondary prevention”, such as installing a sprinkler system, the former option is a safer alternative, all else being equal. This is because the sprinkler system might fail through some unfortunate sequence of events, or be destroyed by a malicious actor, in which case a major fire might occur, while this is ruled out entirely if the hazard is removed (Hansson, 2010). Advocates of decentralised systems can be understood as arguing that their preferred systems are inherently safe (“privacy preserving by design”), while centralised systems are not. In centralised systems, we have to trust that the information on the central server can be adequately protected against breaches (Ahmed et al., 2020), and in order to underwrite this trust, legislation would need to be enacted and strictly enforced that would prevent information on the server from being accessed and used for foreign purposes (for instance, by law enforcement).^4^ But, being secondary interventions, these regulations cannot entirely exclude risks of breaches. These breaches might reveal the *social graph*, that is, a graph that depicts social ties between users (Troncoso et al., 2020). In contrast, “decentralised systems have no distinct entity that can learn anything about the social graph” (Joint Statement, 2020). By removing the hazard entirely, these systems presumably rule out the risk of breaches and are inherently safe.

The argument that centralised systems entail risks of breaches, while decentralised systems rule out such risks, has been influential in debates about digital contact tracing and was part of the reason that centralised apps have fallen out of favour in many places. For instance, the European Parliament resolution on EU coordinated action to combat the COVID-19 pandemic and its consequences demands that “the generated data are not to be stored in centralised databases, which are prone to potential risk of abuse and loss of trust and may endanger uptake throughout the Union… all storage of data [must] be decentralised” (European Parliament, 2020). However, as engineers are well aware, inherent safety is not always possible as it may reduce the likelihood that a given design achieves its purpose (Möller & Hansson, 2008). We have suggested, thus far, that this might be such a case—it is the very collection of this information that decentralised advocates worry could be unmasked to reveal the social graph that allows centralised servers to identify whether clusters result from infections, and thus opens up the possibility of allowing for more rapid reporting. It seems, then, that failing to collect this information, even if it were to inherently protect the system against privacy breaches, might undermine the efficiency of the system, leaving it less able to achieve its purpose: controlling of the spread of infection. It is thus not clear, in this particular case, that inherent safety is to be preferred.

## Evaluating Ethical Risks: Probability and Severity

But there is also a second problem here—cryptographers have cast doubt on the claim that decentralised systems are in fact inherently safe, as it is not central storage of data alone that entails risks of breaches (Ahmed et al., 2020; Vaudenay, 2020). Rather, they argue that different systems entail risks of *different kinds of breaches*. While a chief concern raised against centralised systems is that a malicious authority might access information on the government-administered, central server and identify users and their contacts, thus revealing the social graph, a problem with decentralised systems is that, because users’ ephemeral identifiers are stored on their phones, access to an individual’s phone would reveal more information than in a centralised system. Vaudenay suggests, for example, that in a decentralised system, “after a burglary during which a Bluetooth sensor captured an ephemeral identifier, suspects could have their phones inspected for two weeks to find evidence” (2020, p.6). Vaudenay notes, in addition, that an individual’s smartphone is much easier to hack than a central server. Furthermore, decentralised systems are more vulnerable to breaches that would identify infected users. This is because all of a user’s ephemeral identifiers are uploaded to a central server when they report as infected. Because these identifiers are accessible to any user of the app, it is possible for any user to identify infected users, by recording a user’s identifiers and later comparing them to the identifiers stored on the server (Tang, 2020).

If neither centralised nor decentralised systems are inherently safe, but rather entail risks for different kinds of breaches, how should these risks be traded off against each other? It might at first glance seem that preventing the risk of a major fire—that is, revealing the social graph—is more important than preventing the risk of small fires—identifying single infected users, and thus that concerning the risks of privacy breaches, decentralised systems are clearly preferable to centralised systems. However, it is not clear whether this is true. According to standard usage in professional risk analysis, “risk” refers to the expectation value of an unwanted event, that is, the product of the probability of that event happening and a measure of its severity, or “disvalue” (Hansson, 2004, p. 10). Adopting this notion for the comparison of the risks of different kinds of breaches, there are two dimensions that must be compared here, namely the probability of the breaches happening, and their severity, respectively.

As to their *severity*, it might appear from outside that the disvalue from revealing the social graph is more severe than that of single infected persons being identified. However, users who are concerned about being identified in this way if infected may come to the opposite conclusion. This is backed up by empirical evidence; a survey conducted by Li et al. (2020) suggests that users are more concerned by the privacy vulnerabilities of a decentralised system, and would be more likely to use a centralised app, based largely on concerns about privacy. In this study (in which, notably, they assumed that users would be directly identifiable by central authorities in a centralised system, rather than being issued a pseudonymous identifier), some of those surveyed considered government authorities trustworthy and were willing to provide their information to them, while expressing concerns about the vulnerabilities of the decentralised system to leak information to tech-savvy individuals. Others expressed concerns about privacy violations in both systems, but regarded the vulnerabilities of decentralised systems as “a more severe threat” (2020, p.20).

How, then, do the *probabilities* of the different kinds of breaches happening compare? In decentralised systems, the probabilities of breaches appear to be high, because any user could in principle identify infected users in the manner described above; in contrast, breaches in centralised systems would be very difficult to achieve, and would probably require a malicious government authority to store additional information as an app user registers, which would make identification possible (Vaudenay, 2020). Thus, concerning the likelihood of breaches, centralised systems may have an advantage over decentralised ones.

Thus, when we compare the risks of the different kinds of breaches, it is not clear whether decentralised systems display an advantage on the severity dimension, while centralised systems exhibit a clear advantage on the likelihood dimension. Equipped with these results, we can now turn to the overall ethical evaluation of the two kinds of digital contact tracing options. There are two substantive kinds of ethical risks involved here: risks for privacy, and risks for public health if the contact tracing effort turns out to be ineffective. Some have argued that the risks for privacy may be acceptable if an app is an effective means of achieving public health benefits (Ranisch et al., 2020; Schaefer & Ballantyne, 2020). These two kinds of ethical risks are, however, difficult to trade off against each other, or might even appear incommensurable, and any particular claim about how they might compare will likely be subject to criticism. Rather than directly comparing the benefits and risks for public health and privacy, we merely draw on the principle that other things being equal, the higher the likelihood of a system being effective in bringing about public health benefits, the higher the level of risks for privacy that should be regarded as acceptable. Thus, because the likelihood of centralised systems being effective is higher, other things being equal, a higher level of risks for privacy may be acceptable. In other words, to possibly outweigh the risks for public health, the privacy risks in decentralised systems would have to be clearly much lower than those inherent in centralised systems, as might be the case if decentralised systems were to fare much better on both risk dimensions—that is, if the severity of possible breaches were clearly lower and their likelihood were lower, too. But we have argued that this is not the case. Because the overall ethical risks from centralised systems are thus lower, they should be regarded as an ethically preferable alternative to decentralised systems.

## Installing a Sprinkler System

Now that we have identified and evaluated the risks in both centralised and decentralised systems, we should return to the notion of secondary prevention measures. We have now established that both centralised and decentralised systems entail risks: neither of these systems are inherently safe when it comes to privacy breaches, and so both will require secondary prevention measures to mitigate privacy risk. Constructing and implementing such measures is certainly no small task for either system. Vaudenay presents a pessimistic view of the possibility of mitigating the propensity of decentralised systems to reveal the identity of infected users, contending that these attacks “are undetectable, can be done at a wide scale, and…proposed countermeasures are, at best, able to mitigate attacks in a limited number of scenarios.” Attacks to centralised systems, on the other hand, he suggests, can be better identified and mitigated by “accounting and auditing” (2020, p.6).

This does not quite tell the whole story; we will need an adequate infrastructure in place to protect the centralised server against misuse of the information it stores, particularly by the government authority that is entrusted with this information. This will require legislation explicitly limiting the type of information that can be collected, and preventing the use of contact tracing data for non-public health purposes, such as that introduced in some US states (New Jersey Department of Health, 2020; New York State Senate, 2020). It requires a robust and independent judicial system that will stringently enforce these requirements (see e.g. the Provincial Court of British Colombia’s 2014 decision about the disclosure of information concerning HIV-positive individuals). In addition, it necessitates agencies that have the autonomy to conduct the kind of “accounting and auditing” of the system that Vaudenay points out could allow us to spot problems.

It should not be expected that these elements will simply fall into place without careful oversight, discussion and planning. But nor should it be assumed that these risks cannot be minimised. Just as many countries allow the collection of (non-pseudonymised) information for manual contact tracing purposes, or some countries allow the centralised storage of digital health records, such a policy should be approached with awareness of the risks and the measures necessary to mitigate them, but not completely taken off the table. It is also noteworthy that, while we have focused on general lockdowns and decentralised digital contact tracing as our points of comparison here, other potential pandemic mitigation measures involve ethical risks too. General lockdowns involve direct health risks and economic damages that may entail further risks for public health. Some other measures have been proposed to overcome lockdowns. For instance, Savulescu and Cameron, (2020) propose a policy of selectively locking down the elderly, while allowing the rest of the population to go about their lives unhindered. However, it has been argued that such a selective isolation policy would severely discriminate against the elderly (van Basshuysen & White, 2020; White & van Basshuysen, 2020). Such discrimination would risk violating another substantive value, namely justice (Ranisch et al., 2020). Given that in a pandemic such as COVID-19, there are no risk-free options, the risks of centralised contact tracing, particularly when we take into account the risks inherent in our alternative options, might indeed be worth taking under these circumstances.

## Conclusion

We have argued that in order to provide us with an appropriate balance between a general lockdown and unrestrained viral proliferation, contact-tracing apps must collect some pseudonymised information about their users on a central server. However, privacy advocates have expressed concerns about such a system, contending that contact-tracing apps should be “privacy preserving by design”, while at the same time arguing that this cannot be achieved by systems that rely on central data storage. According to this line of argument, only decentralised systems can be designed to preserve privacy, as these systems can preclude breaches by storing very little data on central servers.

We have evaluated this argument by drawing on a principle from safety engineering—that we should typically strive to make a design inherently safe, rather than merely reducing the likelihood of potential hazard through secondary prevention. We argued, however, that decentralised systems are not inherently safe (i.e. fail to be “privacy preserving by design”), primarily because these systems broadcast the ephemeral identifiers of infected users, which can be used to identify these users. After showing that both systems entail privacy risks, we conducted an assessment of the overall ethical risks of centralised and decentralised systems, taking into account that digital contact tracing options may not only involve risks for privacy, but may also involve considerable risks for public health if they fail to allow for effective contact tracing. While trading off the risks for privacy against those for public health would be difficult and any particular claim about how these risks might compare might be disputed, our argument does not rely on a comparison of these two kinds of risk. Rather, we argued that, if the likelihood of a system being effective is higher, other things being equal, a higher level of risks for privacy should be regarded as acceptable. Because decentralised systems have a smaller chance of being effective, it follows that their ethical risks would only compare favourably to centralised systems if the latter were to entail privacy risks that are clearly much higher than those of decentralised systems. This might be the case if decentralised systems were to fare much better on both risk dimensions—that is, if the severity of possible breaches were clearly lower and their likelihood were lower, too. We argued, however, that this is not the case. It follows from this risk assessment that, all things considered, centralised systems should be seen as involving less overall ethical risk than decentralised systems, and may thus be the ethically preferable option.

Where does this leave us with respect to ethically justifiable policy making concerning digital contact tracing, and the technological use of personal data more generally? The privacy advocates’ arguments have been influential in debates about digital contact tracing and, backed by Apple and Google’s strategy to make it difficult to produce a centralized app which can function effectively on their smartphone systems, they have apparently led policy makers in most countries to implement decentralised systems. It follows from our risk assessment that these policy makers may well have been *mis*led, as centralised systems are in fact the option that could minimise overall ethical risks. The trajectory of this debate provides us with reason to be cautious about allowing privacy concerns to trump other relevant values. Others have pointed to further problems with focusing solely on data privacy to the neglect of other ethical issues, both in the context of contact-tracing apps (see Sharon, 2020) and in other uses of data (e.g. Prainsack & Buyx, 2016). By conducting an overall risk analysis, we can widen our focus to incorporate other considerations and draw them into our ethical assessment of data technologies.^5^

## Footnotes

1 It should also be noted that increasing the speed and thus efficiency of the app might also compensate to a certain degree for lower uptake, allowing for outbreak control without requiring an unrealistically high proportion of the population to use it (see Hernández-Orallo et al., 2020).

2 Often by issuing them with a code which they must enter into their app when submitting a positive report, see e.g. Dillet, 2020; Robert Koch Institute, 2020.

3 The collection of these data on a central server may have further advantages, for instance, as input for epidemiological research or public health care planning (Lucivero et al., 2020). Centralised storage is thus also conducive to important public health values.

4 We will return to this point presently.

5 Many thanks to an anonymous reviewer, whose helpful comments allowed us to significantly improve the manuscript.
